# Advances in light system engineering across the phototrophic spectrum

**DOI:** 10.3389/fpls.2024.1332456

**Published:** 2024-02-12

**Authors:** Galen Dennis, Matthew C. Posewitz

**Affiliations:** Department of Chemistry, Colorado School of Mines, Golden, CO, United States

**Keywords:** photosynthesis, light reactions, cyanobacteria, plants, microalgae, genetic engineering

## Abstract

Current work in photosynthetic engineering is progressing along the lines of cyanobacterial, microalgal, and plant research. These are interconnected through the fundamental mechanisms of photosynthesis and advances in one field can often be leveraged to improve another. It is worthwhile for researchers specializing in one or more of these systems to be aware of the work being done across the entire research space as parallel advances of techniques and experimental approaches can often be applied across the field of photosynthesis research. This review focuses on research published in recent years related to the light reactions of photosynthesis in cyanobacteria, eukaryotic algae, and plants. Highlighted are attempts to improve photosynthetic efficiency, and subsequent biomass production. Also discussed are studies on cross-field heterologous expression, and related work on augmented and novel light capture systems. This is reviewed in the context of translatability in research across diverse photosynthetic organisms.

## 1 Introduction

In 2022 the world population reached 8 billion. The United Nations Population Division projects it will grow to 9 billion by 2037. Although the rate of population growth is slowing, it will likely exceed 10 billion by 2100 (United Nations). This growth represents a staggering increase in demand for food, fuels, and useful materials, yet there already exist considerable populations across the globe with limited resources in one or more of these categories (U.N. Environment, 2019). Pressures produced by increased resource demands are exacerbated by the growing consumption of limited materials such as fossil fuels and the increased production of pollutants and greenhouse gases (Daly and Farley, 2011). In addition, sources of freshwater are simultaneously being used and polluted at an unsustainable rate, driven by contemporary agriculture (Hoekstra and Mekonnen, 2012; Kummu et al., 2016; Shi and Tanaka, 2020).

Biomass from phototrophic growth is a promising component of the solution to these issues through its use as a source of food (Chacón-Lee and González-Mariño, 2010; Tilman et al., 2011; Ort et al., 2015), fuel (Ort et al., 2015; Bhat et al., 2022) and materials (Rehman et al., 2017, p. 20; Toker-Bayraktar et al., 2023). Utilizing biomass has several environmental advantages as it is a sunlight driven process, and fixes the largest greenhouse gas contributor, CO_2_. However, there are limitations to how much biomass is currently produced through photosynthesis. Some current bottlenecks include the photosynthetic process itself, and technological limitations in harvesting and processing biomass, as well as extracting useful materials. Most commercial biomass sources are from terrestrial plants, so the availability of conventionally arable land and freshwater is another important consideration (OECD, 2011). In addition, plant evolution has led to limited genetic diversity when compared to cyanobacteria or microalgae (Häder, 2022). On the other hand, much work is focused on making cyanobacterial and microalgal cultivation practical at the large scales needed to effectively complement traditional agriculture (Novoveská et al., 2023). It is possible that cultivation of microalgae can bypass or mitigate these concerns by production in non-arable environments using non-freshwater sources (e.g., saltwater, wastewater) (Ahmad et al., 2022; Energy.gov).

**Figure 1:**
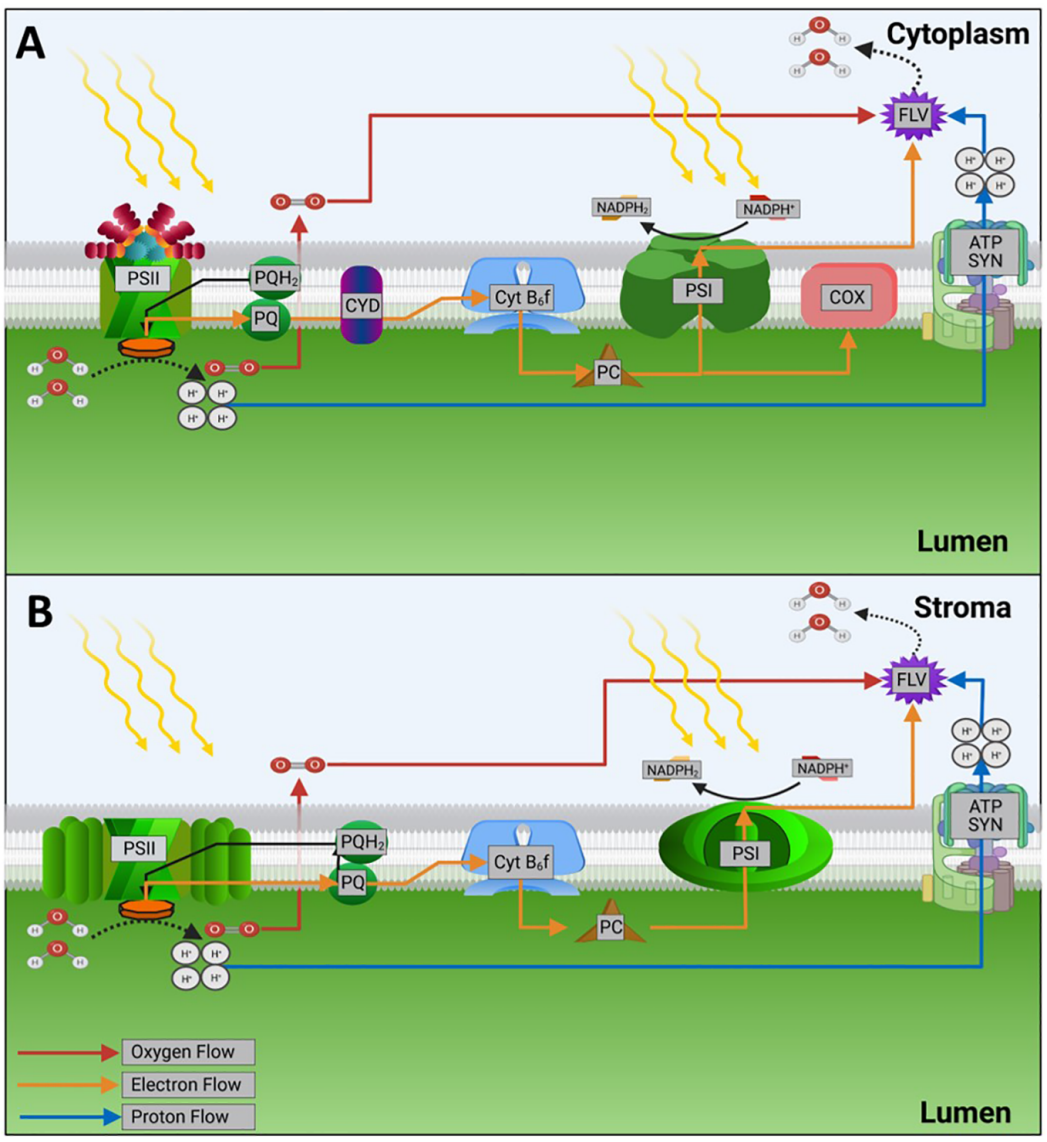
The fate of light energy in photosystems. Orange arrows represent electron flow. **(A)** Representative cyanobacterial photosystem. **(B)** Representative eukaryotic photosystem. Note that only those system components relevant for this review are shown. Abbreviations are as follows: PSII-photosystem II, PQH_2_/PQ-plastoquinol/plastoquinone, Cyt *b_6_f*-cytochrome *b_6_f* complex, PC-plastocyanin, PSI-photosystem I, FLV-flavodiiron protein, ATP SYN-ATP synthase, COX-cytochrome *c* oxidase, CYD-cytochrome *bd*-type quinol oxidase. Figure made with bioRender software, based on Nikkanen et al., (Nikkanen et al., 2021).

For the purposes of this paper phototrophic biomass is delineated into three sources: cyanobacteria, eukaryotic microalgae, and terrestrial plants. Each system has a practical advantage in terms of sustainability. Plants are the current industrial standard for bulk bioproducts (e.g., palm oil) and staple food production. The rapid growth times and relatively simple metabolic systems of these microorganisms are promising for both fundamental and applied studies to improve photosynthetic efficiency and rationally engineer productive strains. When considering photosystems, eukaryotic plants and algae are more similar to each other than cyanobacteria (**Figure 1**). However, in terms of physiology, eukaryotic microalgae share more in common with cyanobacteria. The combination of phototrophic bacteria, eukaryotic microalgae, and terrestrial plants represents an impressively diverse set of organisms and expands the possible resources and environments which can be utilized in making biomass. In this manuscript, we present a wide range of organisms and genera by describing recent publications related to engineering their photosystems. For the sake of brevity only a brief introduction to each organism is included, and the reader is encouraged to read the presented papers for more detailed descriptions.

**Figure 2:**
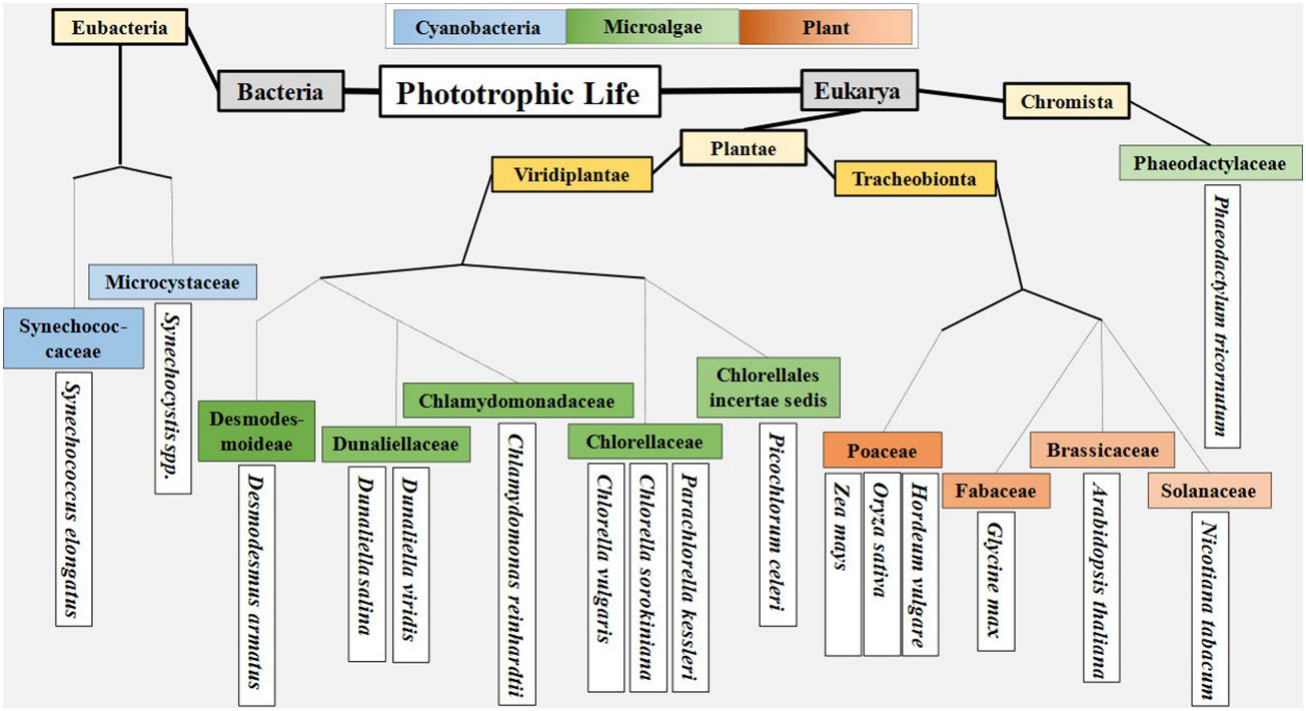
Delineation of organisms that have recently been studied in context of photosynthetic light reactions. Taxonomy for cyanobacteria and microalgae obtained from algaebase.org. Taxonomy for plants obtained from the USDA Plant Database.

The diversity between cyanobacteria, microalgae, and plants is best looked at through the evolutionary lineage of photosynthetic organisms, which began in bacteria, and continued to eukaryotic microalgae, and then to plants (Häder, 2022). Delineating along these three lines provides a convenient way to categorize phototrophs. In terms of light reactions, recent work has focused around a relatively narrow set of organisms (**Figure 2**). Plant related work has focused on the model systems *Arabidopsis thaliana* and *Nicotiana tabacum* (tobacco), and staple crops such as soy (*Glycine max*), rice (*Oryza sativa*), barley (*Hordeum vulgare*) and maize (*Zea mays*). Microalgal work centers around *Chlamydomonas*, *Chlorella*, *Dunaliella*, and *Phaeodactylum*, with additional work being done in emerging systems of interest such as *Picochlorum, Desmodesmus*, and *Parachlorella*. While most work in cyanobacteria has been done with strains of *Synechococcus* and *Synechocystis*. The study of each of these organisms presents a broad range of physical and genetic diversity that serves to push our understanding of genetic engineering in oxygenic photosystems.

Several detailed reviews of all three fields, cyanobacteria (Vijay et al., 2019; Luan et al., 2020; Mehdizadeh Allaf and Peerhossaini, 2022), microalgae (Vecchi et al., 2020; Hu et al., 2023), and plants (Nowicka, 2019; Miglani et al., 2021; Theeuwen et al., 2022; Kumar et al., 2023; Leister, 2023; Li et al., 2023), were published within the past five years. Many authors focus on the parallels between plants and eukaryotic algae (Kirst and Melis, 2014; Guardini et al., 2022; Sharma et al., 2023), with relatively little focus on understanding parallels between microalgae and cyanobacteria (Wijffels et al., 2013). There is an apparent lack of work comparing all three systems simultaneously, despite similar ideas for how to improve photosynthetic efficiency, particularly depigmentation, improved oxidative stress resistance and enhanced electron sinks. The respective benefits of modifying these systems are controlling the initial photon flux into the system, reducing uncontrolled electron transport, and providing regulated dissipation of excess energy absorption. However, as several of the above reviews point out, techniques used for one species to improve photosynthesis can yield contrasting results in different species.

Much of the work related to expanding and augmenting photosystems to increase the photosynthetic action spectrum centers around cyanobacteria. This is an emerging field, with most work being done within the last decade. Chlorophyll *d* is a cyanobacterial pigment that can replace chlorophyll *a* in the photosystem I (PSI) reaction center, driving photosynthesis at infrared wavelengths (Chen and Blankenship, 2011; Allakhverdiev et al., 2016). Similarly, several studies have shown that pigments absorbing in ‘unused’ wavelengths (e.g., green light) can be integrated as additional photosynthetic pigments into light systems (Leister, 2023). In terms of application of these systems, Hitchcock et al. recently published an overview of a consortium project called *PhotoRedesign*, which is attempting to expand the working light spectrum in photosynthetic organisms (Hitchcock et al., 2022). This is an appropriate addition in this review because these novel light systems are being integrated *de novo* into photosystems of all three organisms.

## 2 Cyanobacterial photosynthesis

### 2.1 Depigmentation in *Synechococcus*

The gene *nblA* was recently overexpressed in *Synechococcus elongatus* by Carrieri et al. This gene codes a protein that disassembles phycobilisomes and is specific to cyanobacteria. Overexpression led to a depigmented mutant strain of *Synechococcus elongatus* PCC 7942. Three mutant lines were tested with high (25-fold), medium (8-fold) and slight (3-fold) increases in gene expression. Gene expression correlated with pigmentation levels. All strains had improved O_2_ evolution over the WT on an OD_750_^-1^ L^-1^ basis, and the medium expression strain showed the greatest improvement. When tested further, this strain had 25% improved biomass accumulation under 1000 µmol PAR constant light. The authors proposed that this was due to reduced photodamage rather than reduced self-shading because the transformed strain dominated in transformant-WT co-cultures. This study provides evidence for the hypothesis that lower pigmentation leads to improved growth due to less photodamage rather than improved light penetration through the culture. In addition, it is an example of an alternative strategy for reducing pigmentation, as opposed to CRISPR mediated pigment reduction (Carrieri et al., 2021).

Adding to this body of work was a study published on phycobilisome rod deletion using CRISPR/Cas3 and CRISPR/Cas12a. The authors targeted the genes *cpcG* and *rrl* in *Synechococcus* 2973. These genes correspond to rod-core linker and rod-rod linker proteins within the phycobilisome rod and knocking out the former resulted in the total loss of phycobilisome rods, while the latter knockout produced truncated rods, with both exhibiting a depigmented phenotype. Under a high light (800 µmol PAR) and high CO_2_ (1%) condition, the *cpcG* edited strain produced about 20% of the chlorophyll compared to the *rrl* and WT strains and approximately 10% of the WT phycocyanin content, whereas the *rrl* edited strain showed no change in chlorophyll concentration and approximately 50% of the WT phycocyanin content. The reported F_v_/F_m_ of the *cpcG* knockout decreased to approximately 20% of the WT, and the *rrl* knockout F_v_/F_m_ increased by about 16%. The authors presented a comparison of the *rrl* knockout and the WT under a variety of light levels and CO_2_ concentrations. In terms of doubling time, the edited line only outperformed the WT under air levels of CO_2_, while the trend reversed or was not present under 1% CO_2_. This suggests reduced photodamage to cells under excess light as photon input and carbon assimilation are better balanced (Sengupta et al., 2023).

### 2.2 Rational engineering and photosystem balance in *Synechococcus*

Two studies have recently been published in various *Synechococcus* strains showing the effects of heterologous cytochrome P450 expression as a sink for excess electrons moving through PSI. The first study examined the combinatory effects of expressing cytochrome P450 as an electron sink through the mono-oxidation of a variety of substrates, and a sucrose synthase and transporter to act as a carbon sink in *Synechococcus* PCC 7942. The authors tested all four permutations of these heterologous proteins and found that sucrose export acts synergistically with cytochrome P450 to improve the overall photosynthetic efficiency of the cell (Santos-Merino et al., 2021). The second study sought to understand how removing the native cytochrome c oxidase (COX) could be used in combination with heterologously expressing cytochrome P450. The logic of this study was that removing COX would increase electron flux through PSI, thus disposing of excess electrons by cytochrome P450 downstream of PSI. After transforming Synechococcus sp. PCC 7002 with cytochrome P450 in a strain with COX knocked out, the authors found higher (up to 5-fold at 200 µmol PAR) activity by cytochrome P450 in the knockout line under light intensities above 24 µmol PAR. The knockout line expressing cytochrome P450 also reached higher saturated electron transport rates than the control strains. (Torrado et al., 2022) For both studies, it is difficult to say how these results would translate in industrially relevant conditions without higher light or diel growth curves and biomass measurements, however they present excellent methods for increasing and controlling electron flow through PSI.

Work published by Ungerer et al. in 2018b, showed that photosystem stoichiometry is important for increasing the growth rate of *Synechococcus elongatus* UTEX 2973 over the nearly identical strain *Synechococcus elongatus* PCC 7942. UTEX 2973 has more electron carriers in the electron transport chain (ETC), specifically cytochrome *f* and plastocyanin. In addition, UTEX 2973 reached 1.6-fold greater PSI complex expression. This translated to a 3-fold higher growth rate, 2.5-fold increase in glycogen content and a corresponding 2.5 times increase in carbon fixation rate (Ungerer et al., 2018a). The implication of this study is that photosystem stoichiometry plays a role in the overall growth rate of an organism, perhaps explaining why studies that specifically target only a single reaction center produce mutants with decreased growth rates (De Mooij et al., 2015; Nymark et al., 2019).

The same group followed this study with a comparative genomic analysis between UTEX 2973 and PCC 7942. In this work, they used mutational mapping to narrow down the ~50 differing genes between the two strains to three associated with rapid growth, which were *atpA*, *ppnK* and *rpaA*. The authors used CRISPR/Cpf1to replace the genes in PCC 7942 with those from UTEX 2973, which led to faster doubling times and biomass accumulation. By replacing all three, the doubling time of strain 7942 was reduced from 6.9 h to 2.2 h, only slightly longer than the UTEX 2973 doubling time of 2.13 h. The function of each gene was proposed to be as follows. ATP synthase AtpA had reduced redox regulation in UTEX 2973 due to the conversion of a surface cysteine to tyrosine. The *ppnK* gene encodes an NAD^+^/ATP kinase, and UTEX 2973 uses an aspartic acid in the active site, as opposed to glutamic acid. This localizes the negatively charged group closer to the substrate, putatively improving the active site kinetics. Finally, the *rpaA* gene encodes an important circadian clock regulator, and UTEX 2973 had both an altered promoter and two mutated amino acids. The authors were able to show that each of these changes were important for improved growth, but their exact effect was not explored (Ungerer et al., 2018b). An important caveat to this study was brought forth in an extensive comparative genomics study on *Synechococcus* strains by Adomako et al. This work sequenced a different clonal isolate of *Synechococcus* UTEX 2973, and found no indication of the *rpaA* mutation, which indicates that this mutation was unique to the isolate studied by Ungerer et al. (Adomako et al., 2022).

### 2.3 Depigmentation in *Synechocystis*

*Synechocystis* is another cyanobacterial model that is well studied. One group removed phycocyanin from *Synechocystis* sp. PCC 6803 by inactivating the *cpc* gene through homologous recombination. This increased the light saturation point of the edited line by 2-fold and biomass accumulation improved at 2000 µmol PAR constant light. The knockout strain showed decreased light absorbance relative to the WT at culture depths between 1 and 5 cm. The depigmented strains showed decreased photosynthetic efficiency at lower lights levels (50-170 µmol PAR), which indicates reduced light harvesting ability due to the lack of phycocyanin. This work shows that the principle of reduced pigmentation improving photosynthetic efficiency applies in cyanobacteria, particularly with reference to the phycobilisomes attached to PSII (Kirst et al., 2014).

### 2.4 Alternative electron flow in *Synechocystis*

Also in *Synechocystis* sp. PCC 6803, Hasunuma et al. over expressed the flavodiiron 3 protein leading to improved alternative electron flow. The transformed strain showed significantly higher biomass accumulation over the WT and plasmid control strains in addition to higher glycogen levels. Oxygen evolution experiments corroborated this and C^13^O_2_ uptake studies of various metabolites in carbon fixation. The authors ended the study by showing that the overexpression of this gene leads to additional NADP^+^ reduction at PSI, which in turn feeds directly into carbon assimilation (Hasunuma et al., 2014). Smolinski et al. published a complementary study to this work by eliminating O_2_ reduction activity by flavodiiron 1 in *Synechocystis* PCC 6803. This was accomplished by knocking out the *flv1* gene flavodiiron 1. The mutant strain, termed ORR1, showed a change in photosystem I activity through attenuated O_2_ reduction and presented a shift in the photosystem arrangement from trimer to monomer, with monomer content increasing between 1.4 and 5-fold. These results indicate that flavodiiron activity has an influence on photosystem arrangement through its role as an alternative electron transport pathway. Although growth capabilities were not measured for the mutant strains, lowered reducing efficiency in P700 was observed, implying that the imbalance between electron generation and utilization at PSI emerged by taking away the electron sink, thus impairing the overall photosynthetic efficiency(Smolinski et al., 2022). This could represent a way to diminish photosystem I action to balance reduced light absorption at photosystem II light harvesting antenna. The combination of these two studies reveals powerful insights into the function of flavodiiron proteins in cyanobacterial systems.

## 3 Photosynthetic efficiency in eukaryotic microalgae

### 3.1 Depigmentation in *Chlamydomonas*

*Chlamydomonas* is an important model system in microalgal research and is used for many studies. The first reported Cas9 mediated Clustered Regularly Interspaced Short Palindromic Repeats (CRISPR/Cas9) knockout in *Chlamydomonas reinhardtii* was done in 2016 (Baek et al., 2016). The authors generated a double knockout strain with smaller antenna and improved photosynthetic efficiency by introducing a targeted frameshift in the *cpFTSY* gene, which in turn prevented full antenna assembly. At the same time, the zeaxanthin epoxidase (*ZEP)* gene was knocked out to prevent the epoxidation of zeaxanthin. This produced a 10-fold increased accumulation of zeaxanthin in the cells. The double knockout and the cpFTSY knockout had three-fold or higher cell densities when grown under 700 µmol photosynthetically active radiation (PAR) constant light, and all knockouts had improved O_2_ evolution up to 1200 µmol PAR.

Although reducing antenna size is generally regarded as an important step in improving photosynthesis, de Mooij et al. found in 2015 that small antenna mutants had decreased high light tolerance (De Mooij et al., 2015). They found that three previously generated depigmented mutant strains did not outperform the wild type in terms of productivity or light conversion efficiency. These mutants showed reduced pigmentation due to incomplete, but still functional, light harvesting complexes (LHCs). This result contrasted both the authors’ expectations, and results found in a variety of other studies (Baek et al., 2016; Caddell et al., 2023; Krishnan et al., 2023). The authors proposed two possible explanations, first that the mutants could have higher susceptibility to photodamage, second that the mutation strategies used resulted in off target gene editing with negative side effects. In a similar vein, Jeong et al. generated LHC protein translocation defect (LTD) knockouts of *C. reinhardtii* with reduced photosystem I (PSI) and LHCI content. This has a parallel in Arabidopsis, where the effect was more severe (Jeong et al., 2018). The authors point out intriguing differences in the phenotypes and link them to differences in the developmental programs of the plant and microalgal cells, where the former grows to a set volume and chloroplast number, and the latter has continuous growth until division with only a single chloroplast. The LTD chaperone protein is specific for eukaryotic organisms as it delivers LHC proteins to the thylakoid membrane. In *C. reinhardtii*, LTD is not critical for viability but does increase LHCI levels. When compared to the WT, the total chlorophyll content was lower, but the *a/b* ratio was not significantly different. The mutant cells appeared to have a reduced growth rate and cell density when grown at low light (at or below 350 µmol PAR), which indicates reduced cell fitness, potentially due to an unbalanced ratio of photosystem II (PSII) to photosystem I, which is also reflected in the reported reduced photosynthetic efficiency.

### 3.2 Photosystem protection and repair in *Chlamydomonas*

A recent study published data on the immunophilin gene *cyn28*, coding a protein designated CYN28, that is critical for high light acclimation in *C. reinhardtii* (Fu et al., 2023). Immunophilins are widespread chaperones for ensuring proper protein folding. CYN28 specifically assists with the formation of protease FtsH complexes, which are subsequently involved in repairing photodamaged PSII reaction centers, specifically the D1 subunit. This repair cycle is especially relevant as it is conserved across plants, microalgae, and cyanobacteria. The authors identified several immunophilin genes that were upregulated in response to high light. They obtained a *C. reinhardtii* mutant with the *CYN28* gene coding a immunophilin knocked out from a *C. reinhardtii* mutant library (Li et al., 2019). This mutant showed drastically reduced photosynthetic efficiency and a correlating loss of PSII reaction center proteins. The authors found that re-expressing the CYN28 gene, even up to as little as 10% of the original WT expression, was enough to restore photoprotection in high light. The authors went into depth exploring this protein, which should be considered when trying to overexpress PSII reaction center proteins.

In another *C. reinhardtii* photoprotection study, a strain expressing the β-carotene ketolase enzyme (BKT) (Perozeni et al., 2020) showed reduced photoinhibition (Cazzaniga et al., 2022). This transgene expression combined optimizing heterologous gene expression in *C. reinhardtii*, codon optimization, and intron splicing. The resulting mutants reached expression titers of 0.26-0.37 mg L^-1^ of astaxanthin, depending on lighting conditions. The authors found increased absorption spectra in several thylakoid components. In addition, the mutant lines had improved O_2_ evolution above 500 µmol PAR, and continued photosynthetic activity up to 2500 µmol PAR, whereas the WT suffered photoinhibition at 1250 µmol PAR. The mutant strain showed significantly reduced (near zero) levels of NPQ across all light intensities. In this case, adding pigments did not reduce the overall photosynthetic efficiency of the culture as the mutant strain achieved higher biomass than the WT in all media and culturing conditions tested, except for a small decline in growth rate at lower (100 µmol PAR) light levels. Going in a different direction, another study showed that reducing the dosage of genes encoding stress related light harvesting complex (LHCSR) proteins, which are responsible for the dissipation of excess excitation energy, termed qE, can reduce NPQ in *C. reinhardtii*. The edited strains did not show improved growth over the WT, and the LHCSR knockouts had significantly lower NPQ at 50, 400 and 1000 µmol PAR. This corresponded with increased singlet O_2_ production over the WT. This illustrates that reducing energy dissipation mechanisms is not by itself guaranteed to improve photosynthetic efficiency in an alga (Barera et al., 2021).

A complementary study was published by Antonacci et al., who fused heterologous antioxidant peptides to the D1 subunit of PSII in *C. reinhardtii*. This provided localized protection from ROS at PSII. The group tested three different antioxidant peptides, sourced from milk, egg yolk and hoki fish. All transformed strains had lower chlorophyll and carotenoid levels when compared to the WT. This effect is not completely explained by the authors, but they suggest it is a side effect of increased oxidative protection as the pigments act less in the role of antioxidant metabolites. In terms of antioxidant activity, each transformant showed survival and growth when exposed to concentrations of Rose Bengal and H_2_O_2_ at 7 µM and 1.3 µM respectively, levels at which the WT died. The transformed lines also had significantly improved stability in F_v_/F_m_ compared to the WT when grown for 113 days. The hoki fish (antiox-f) antioxidant line in particular showed almost no decline in F_v_/F_m_ over the entire time course. The anitox-f line also showed improved stability under multiple days of constant high light (1200 µmol PAR) or high temperature (40°C). This is an interesting approach for improving the robustness of photosynthesis under stressful environments (Antonacci et al., 2021). However little explanation was provided for the exact mechanism of action of the peptides, specifically whether they act as sacrificial or catalytic ROS scavengers. This is critical for understanding how the fused peptides can provide effective ROS protection and would inform any future studies attempting to replicate or extend these results.

### 3.3 Depigmentation and oxidative stress resistance in *Chlorella*

*Chlorella* is another well-known microalgal system, with potential for cultivation at larger scales. Dall’Osto et al. generated a set of mutants from *Chlorella vulgaris* with ethyl methane sulfonate (EMS) mediated mutagenesis. From this they isolated a mutant designated PG-14 with a 50% chlorophyll reduction relative to the WT. This mutant showed increased growth and photosynthetic efficiency relative to the WT at high light. After adaptation to oxidative stress with Rose Bengal, a strain was isolated that had a 68% improvement in biomass yield relative to the WT. The authors suggested that the mutated strains had enhanced capacity for NPQ and PSII repair (Dall’Osto et al., 2019). This is an example of how mutagenesis followed by selective pressure can be used in series to generate improved strains. Another example of using EMS chemical mutagenesis to generate low chlorophyll *C. vulgaris* mutants was published by Schüler et al. in 2020. The most depigmented mutant had a 180-fold decrease in chlorophyl content, leading to almost negligible amounts of chlorophyll. This mutation was viable due to heterotrophic cultivation. Another mutant with a chlorophyll content decrease could be scaled with minimal growth penalty when grown heterotrophically in 200 L fermenters. The authors reported improved nutritional composition in the mutants, making it of some interest for industrial cultivation (Schüler et al., 2020).

### 3.4 Photoprotection in *Chlorella*

Beyond reducing chlorophyll titers, another study showed that increasing carotenoid content in *C. vulgaris* improved biomass production under high light. The two mutant lines tested had a greater than 2-fold decrease in chlorophyll content per cell, with a corresponding reduction in PSII antenna size. However, carotenoid content increased significantly, ranging from a 1.14 to 1.80-fold increase, with no significant change in F_v_/F_m_. Through P_max_ measurements, they determined that the mutants had increased effective quantum yield of photosynthesis. This translated to a 23% improvement in growth rate over the WT when grown at 1400 µmol PAR. The authors pursued whole genome sequencing that revealed a set of seven potential mutations that may work singly or in tandem to produce the pigment phenotype, in addition to a set of six transcription factors that could have mutated (Guardini et al., 2021).

Two recent studies investigated mechanisms of photodamage and photoprotection in *Chlorella.* Bashir et al. showed that singlet O_2_ directly damages the PSII complexes of *Chlorella sorokiniana*, as opposed to the repair or assembly mechanisms of the complex. This is a step forward in clarifying the exact mechanisms of PSII photodamage (Bashir et al., 2021). Grolomoni et al., characterized the violaxanthin de-epoxidase (VDP) enzyme in *C. vulgaris* to better understand the xanthophyll cycle in this species, and therefore components of NPQ. They found that the enzyme was functionally similar to plant VDP enzymes (Adams et al., 1990), and in contrast to work done in *C. reinhardtii*, this enzyme is a critical part of NPQ enabling *C. vulgaris* to survive high light conditions. This illustrates the genetic diversity present in microalgae and reinforces the need for studies beyond one or two model systems (Girolomoni et al., 2020).

### 3.5 Pigment engineering in *Dunaliella*

*Dunaliella* is a genus of industrial interest due to its impressive salt tolerance and high β-carotene production. In 2019, a group expressed β-carotenoid ketolase (*bkt*) and β-carotene hydroxylase (*crtr-B*) genes from *Haematococcus pluvialis* in *Dunaliella viridis* to allow the production of astaxanthin. These two enzymes act sequentially on β-carotene to produce astaxanthin with the order of action depending on localization within the cell. The transformed line had a reduced growth rate compared to the WT when grown under a 12:12 light/dark cycle using low light (60-70 µmol PAR) but had between 20 and 60 µg g^-1^ dry weight of canthaxanthin and astaxanthin. These levels increased by between 1.6- and 1.8-fold when cultured under high light (260-290 µmol PAR). This implies that the two carotenoids were differentially expressed in response to high light stress in the culture with no additional genetic modifications (Lin et al., 2019).

An example of limited rational engineering in *D. salina* was published by Hu et al. in 2021. Using CRISPR/Cas9 for the first time in this genus, the group successfully edited the β-carotene hydroxylase gene (*dschyb*), which is responsible for the conversion of β-carotene to zeaxanthin. A practical result of this work could be reduced NPQ through the VAZ cycle by the edited cells; however, no physiological characterization was done beyond the comparison of pigment and gene expression levels between the knockout strains and WT. The results showed somewhat variable β-carotene and zeaxanthin expression between four different edited lines. Some lines produced equivalent amounts to the WT, and others reached up to three-fold higher β-carotene or half as much zeaxanthin after 24 hours of high light treatment (6000 Lux m^−2^ s^−1^). These results show that zeaxanthin expression was not completely silenced, which implies potential alternative genes or incomplete knockouts. The authors did not explore any possible causes, nevertheless, this is a step forward in gene editing in this species (Hu et al., 2021).

### 3.6 Depigmentation in *Phaeodactylum*

*Phaeodactylum tricornutum* is a model diatom (*Bacillariophyceae*), which are highly divergent from green microalgae, yet remain ecologically and industrially relevant (Benoiston et al., 2017; Sharma et al., 2021). Agarwal et al. explored the function of the MYB transcription factor family by inhibition using antisense ribonucleic acid interference (RNAi). They found that reducing the transcription factor activity led to significant changes in LHC formation and chlorophyll content in *P. tricornutum* in both low light (20 µmol PAR) and high light (940 µmol PAR). When compared to the WT, the transformant with the most stable phenotype had a reduced growth rate and higher chlorophyll in high light, whereas in low light the trend was reversed. This indicates that the transcription factor plays a role in LHC formation in response to light. It is likely that this transcription factor family has orthologs in most eukaryotic algae, such as *Nannochloropsis gaditana* (Agarwal et al., 2022).

As found with pigment knockouts in other species, decreasing light harvesting antenna can impair growth in *P. tricornutum*. Nymark et al. found that loss of the *alb*3b gene decreased pigment binding proteins. This gene is a part of the chloroplast signal recognition particle (CpSRP) pathway. Knocking it out reduced both LHCI and LHCII antenna sizes due to the unique putative property of diatom antenna feeding both reaction centers. Knocking out this gene changed the cell color from brown to green due to a 75% loss of fucoxanthin-chlorophyll *a/c* binding proteins, where fucoxanthin and chlorophyll c are responsible for the brown coloration. Both PSII and PSI showed reduced absorption cross sections, and NPQ was significantly reduced. In terms of O_2_ evolution, this translated to a higher P_max_ on a chlorophyll basis, increasing from 58 to 63 µmol O_2_ mol Chl^-1^ s^-1^ at 35 µmol PAR, and from 55 to 72 µmol O_2_ mol Chl^-1^ s^-1^ at 200 µmol PAR. This increase was not observed when O_2_ evolution was normalized to cell count, which suggests that the cells maintained the WT photosynthetic rate despite lower chlorophyll. The knockout lines had an approximately 2-fold decrease in growth rates at lightings of 200 and 480 µmol PAR, with no difference at the low light levels of 35 µmol PAR. The authors proposed that the reduced growth rate of the knockouts was likely due to a reduced ability to harvest light (Nymark et al., 2019).

### 3.7 Photoprotection in *Phaeodactylum*

Another study of interest describes a protein that provides thermal dissipation for photoprotection in *P. tricornutum* (Buck et al., 2019). Specifically, these authors examined the effect of knocking out *Lhcx* genes on the qE portion of NPQ. The *Lhcx* genes encode diatom specific proteins providing photoprotection via thermal dissipation, but have analogs in the green algal literature, *Chlamydomonas* specifically (Girolomoni et al., 2019). The authors used Transcription Activator-Like Effector Nucleases (TALENs) to generate homozygous knockouts of four different *Lhcx* genes to understand how each functioned. Using a knockout line with no qE, they systematically expressed each version of Lhcx, and found that qE function was restored after adding back only a single gene. In addition, they found that the extent of qE was directly correlated with xanthophyll cycle activity. The authors found no clear answer as to whether one version of each gene is more effective at qE, but instead speculated that multiple versions are maintained by the cell to allow a dynamic response to a wider range of abiotic factors (Buck et al., 2019).

### 3.8 Standalone studies in microalgal light systems

Outside the above model organisms, work emerges regularly on other microalgal strains of industrial interest. Recently Krishan et al. found that the pigment content of *Picochlorum celeri* can be heavily reduced by CRISPR/Cas9 gene editing (Krishnan et al., 2023). This work builds off previous studies done with *Picochlorum celeri* (Krishnan et al., 2020; Cano et al., 2021), and focuses on the cpSRP43, LHCA6, and LHCA7 proteins. The authors generated triple knockout lines with an increased chlorophyll *a/b* ratio and absorption cross sections on a chlorophyll basis and a decrease in total chlorophyll content. Using photobioreactors to model outdoor diel cultures, they showed that one strain could grow at an extrapolated productivity of 50 g m^-2^ day^-1^, a 20% increase over the WT strain.

A second study on *Desmodesmus armatus* sheds new light on the previously documented low NPQ of this organism. They showed that alternative electron flow (AEF) through a water-water cycle compensates for the lack of NPQ and allows growth in light conditions up to 2000 µmol PAR. AEF occurs by reducing O_2_ to water using excess electrons in high light and can be monitored by measuring changes in ^18^O_2_ concentration through membrane-introduction mass spectrometry (MIMS). No genetic engineering was done in this study, but it shows that AEF can be as significant a contributor to photoprotection as NPQ, and should be considered when evaluating and engineering organisms (Ware et al., 2020).

Finally, a recent study showed that elevated trehalose phosphate phosphatase (TPP) relieves high light stress in *Parachlorella kessleri.* This effect was achieved through a reduction in antenna size in high light, and increased hydrogen peroxide reduction. The TPP gene from *Saccharomyces cerevisiae* was heterologously expressed in *P. kessleri.* Three distinct transformants were examined with one showing 35% greater productivity under constant 1000 µmol PAR. This trend was also observed under higher light, in addition to reduced ROS levels (Rathod et al., 2023). The combination of reduced antenna size and increased resistance to ROS is intriguing, and likely connected. However, it should be noted that the improvement in biomass is only demonstrated with cells growing under non ideal conditions, so it is not clear if TPP expression improves photosynthetic efficiency under less stressful conditions. In addition, the authors do not provide clear statistical data on the transformants and WT productivities (e.g. standard deviations), so further work is needed to understand the overall benefits of heterologous TPP expression in microalgae.

## 4 The plant parallel

### 4.1 Depigmentation and photosynthetic efficiency in *Arabidopsis*

Flavodiiron protein expression is also relevant in eukaryotic systems, as shown by a recent study exploring cyanobacterial flavodiiron expression in the C3 model plant *Arabidopsis thaliana*. Tula et al. found that the heterologous expression of two flavodiiron proteins (*flv1* and *flv3*) from *Synechocystis* PCC 6803 in *A. thaliana* increased plant growth under varying light intensities. The transformed plant strains showed improved growth under higher light conditions (600 µmol PAR) in particular. This led to faster growth and greater biomass accumulation and seed production. The authors proposed that the FLV proteins acted as a sink for excess electrons from PSI, enabling a water-water cycle for the plant (Tula et al., 2020).

Three additional light system studies have been published studying *A. thaliana.* In 2016, Jin et al. found a protein related to RNA recognition that is important for photosynthetic efficiency. The gene was termed High Photosynthetic Efficiency1 (*HPE*1). Upon generating knockout strains of this gene, the researchers found lower chlorophyll content and increased chlorophyll *a/b* ratios. They determined that it facilitates signaling between the nucleus and plastid. Its overall effect is a stimulation of biomass accumulation, and overexpressing this gene may be an effective approach for improving photosynthesis (Jin et al., 2016). In a quest to better understand the effect of antenna size on PSII action, Bielczynski et al. studied a mutant line of *A. thaliana* with a 60% loss in the LHCb1 and LHCb2 antenna proteins of the system. An interesting phenotype in this mutant was that it did not compensate for smaller antenna by expressing more reaction centers, leaving it in a permanently depigmented state (Bielczynski et al., 2020). It is worth noting that *Nicotiana tabacum* has literature with similar results (Gómez et al., 2018).

Finally, a study was published in 2020 on overexpressing the NPQ related enzymes violaxanthin de-epoxidase (VDE), PsbS, and zeaxanthin epoxidase (ZEP). This study used the VDE-PsbS-ZEP (VPZ) overexpression construct published in a study on *Nicotiana* (Kromdijk et al., 2016). VDE and ZEP are involved in the VAZ cycle and PsbS is related to non-photochemical quenching. Despite showing higher NPQ under high light and faster relaxation when transitioned to darkness, all the mutants had equal or lower biomass accumulation when compared to the WT under the conditions tested. The authors suggested that the poor growth was due to overprotection by NPQ reducing the amount of light energy directed to carbon fixation (Garcia-Molina and Leister, 2020). This study can be compared to a more recent study in *Glycine max* described below which showed contrasting results (De Souza et al., 2022).

### 4.2 Depigmentation and photoprotection in *Nicotiana*

Two fundamental studies on the model organism *Nicotiana tabacum* have illustrated methods for improving photosynthesis. Kirst et al. found through the analysis of previously generated depigmented mutants of *N. tabacum* that a reduction in chlorophyll can be accompanied by reduced cell count per leaf and greater air space in the spongy tissue. In addition, they found the chlorophyll *a/b* ratio increased from 3:1 to 8:1, and the carotenoid content decreased by 15%. A result of this depigmentation was an increase in the ratios of PSII to PSI from 1.08:1 to 2.93:1, which the authors posited was compensation for the smaller antenna size of photosystem II. The mutated strain saturated at a higher intensity relative to the WT (635 vs 425 µmol PAR, respectively). Finally, they measured a 25% increase in the mutant’s biomass accumulation when grown in a greenhouse (Kirst et al., 2017). Another approach to more efficient photosynthesis was demonstrated by Kromdijk et al. who heterologously expressed VDE, PsbS and ZEP in *N. tabacum*. This led to more efficient NPQ, which in turn generated a 15% improvement in biomass production by allowing a faster transition out of a photoprotected state when set in the shade (Kromdijk et al., 2016).

### 4.3 Depigmentation and photoprotection in *Glycine*

*Glycine max* (soybean) is an important staple crop and protein source, a study was recently published showing the effects of over expressing the VAZ cycle related enzymes VDE, PsbS, and ZEP in the organism. This work replicated and further examined the studies on *N. tabacum* by Kromdijk et al. and *A. thaliana* by Garcia-Molina et al. as described above. While the *A. thaliana* study showed no increased biomass accumulation, this study achieved increased seed yield in 5 out of 8 independent transformants generated during the first growing season. All five lines showed equivalent NPQ to the WT under high light (2000 µmol m^-2^s^-1^ photon flux density (PFD)) but reached a lower steady state NPQ upon transitioning to low light (200 µmol m^-2^s^-1^ PFD). The quantum efficiency of carbon fixation, photosynthetic efficiency and NPQ were measured on WT and transformed lines grown outdoors. Little difference in any of these variables was measured under constant light measurements, however lower NPQ was measured for all transformants under fluctuating light, while the quantum efficiency of carbon fixation and electron transport increased relative to the WT (De Souza et al., 2022).

*G. max* has also been well studied in the context of lowering chlorophyll titers. In 2018, Walker et al. conducted an applied study on chlorophyll content and growth on a set of 68 mutants of *G. max*. Some of the results reported by the authors are applicable to plant systems only, such as leaf reflectance, absorbance, and transmittance (L_R,_ L_A,_ and L_T,_ respectively). However, a key takeaway comes from a comparison made between the truncated antenna mutant *Y11y11* and the WT. The mutant line did indeed have higher photosynthetic activity lower in the canopy but had a slight decrease in photosynthetic activity throughout the entire canopy. The authors concluded that lower chlorophyll titers do not result in more even absorbance throughout the plant canopy, however canopy chlorophyll can be drastically decreased without substantial losses in productivity (Walker et al., 2018). It should be noted that the authors made this model based on the comparison of *Y11y11* to the WT, which has been characterized in earlier papers (Pettigrew et al., 1989; Slattery et al., 2017). Therefore, it is unclear if the conclusions drawn can be extended to other mutants, or beyond the species.

Based on this study, Acebron et al. recently published work on differences in NPQ in a chlorophyll deficient mutant compared to a green strain of *G. max*. The plants were grown in growth chambers under a diel script reaching a maximum of 650 µmol PAR. The authors specifically studied the effect of fluctuating but non-phoinhibitory light. The results showed that the low chlorophyll strain had decreased NPQ and greater F_v_/F_m_ but showed an overall loss in fluorescent yield across the leaf. In addition, the authors found that photosynthesis took 10-20 minutes longer to induce in the mutant, possibly explaining discrepancies in biomass production. This study provides useful data on the energy partitioning of the chlorophyll mutant, however genomic and proteomic data would be a useful complement to work towards rational light system design (Acebron et al., 2023).

### 4.4 Pigment engineering in *Oryza*

*Oryza* (rice) has had many photosynthesis-related publications recently. Jang et al. found a transcription factor in a double haploid cross of two *Oryza sativa* strains that stimulates chlorophyll production (Jang et al., 2022). Labeled OsbHLHqq11, this is in the basic Helix-Loop-Helix protein superfamily and has a single binding domain. The researchers reported close homologs in a variety of other grain species and state that it targets the formation of thylakoids, rather than chlorophyll synthesis. Specifically, the transcription factor interacted with proteins involved in iron supply to the chlorophyll synthesis chain. Another gene discovery study was published by Liu et al., who used a collection of 225 *O. sativa* accessions to conduct a genome-wide search for genes related to chlorophyll expression and regulation. By doing this they were able to identify two genes, one coding a peroxisomal protein whose expression correlated with increased PSII photosynthetic yield and another kinase with strong correlation to NPQ (Liu et al., 2023). Another group recently reported three separate gene knockouts in *O. sativa that* were generated using CRISPR/Cas9. These genes were CpSRP54a, CpSRP54b, and CpSRP43. The first and third are shared among eukaryotic photoautotrophs, while the second is unique to monocots. The resulting knockout lines had decreased chlorophyll content and shorter plant height, in addition to higher photosynthetic efficiency (Caddell et al., 2023).

In another example of how decreasing chlorophyll can have a negative impact if photosynthetic balance is not maintained, one group used RNAi mediated suppression to reduce chlorophyll synthesis in *Oryza sativa* by targeting the *YGL1* gene, which codes an enzyme in the chlorophyll synthesis pathway. Hetero- and homozygous transformants were grown outdoors, and biomass accumulation and photosynthetic efficiency were compared directly with the WT. The homozygous mutants showed low *YGL1* gene expression, less NPQ, higher photosynthetic efficiency, reduced plant height and smaller antenna. The heterozygous lines showed a phenotype in between these two levels. One important result was accumulation of the chlorophyll precursor protoporphyrin IX, which the authors proposed acts as a light sensitizer, and led to increased photodamage under high light. This illustrates the need to consider the over accumulation of precursors if chlorophyll synthesis is impaired (Mao et al., 2023).

### 4.5 Chlorophyll synthesis in *Zea* and *Hordeum*

*Oryza, Glycine, Nicotiana* and *Arabidopsis* represent most light reaction focused research in recent years. However, two studies on other species were also published. Xue et al. found a gene labeled *zm00001d008230*, coding an ester cyclase in *Zea mays* (maize). Using EMS to generate mutants, and then selecting for a pale green leaf phenotype, they found a mutant with decreased pigment content and an increased chlorophyll *a/b* ratio. Using RNA-seq, they found a cyclase gene labeled ZmCRD1, and verified its function as critical in chlorophyll synthesis by homozygous knockouts and complementation. By heterologously expressing a GFP tagged fusion protein and WT proteins in *Arabidopsis thaliana* they showed that mutating the protein leads to significantly altered chloroplast morphology. Overall, the mutant had poor photosynthetic efficiency compared to the WT despite an increase in F_v_/F_m_. The authors proposed that the morphological change in the chloroplast indicates that the enzyme is critical for chloroplast formation and the synthesis of photosystem related products (Xue et al., 2022).

Another group published a chlorophyll synthesis study on a mutant of *Hordeum vulgare* (barley) with a 50% decrease in chlorophyll content. The mutant had improved photosynthetic efficiency at all canopy levels and a lower NPQ fraction across all light levels. The total NPQ was also lower than the control at 500 µmol PAR and above, with a correlating decrease in VAZ cycle pigments from 132 to 83 pmol mg^-1^ fresh weight. This phenotype was traced back to a stop codon rendering the *hvcpSRP43* gene inactive, preventing the accumulation of light harvesting pigments in PSII. As seen before, this did not translate to higher biomass under field conditions, but it does represent a gene that can be targeted when balancing chlorophyll attenuation and photosynthetic efficiency in plants (Rotasperti et al., 2022).

## 5 Augmenting natural light capturing systems

Novel light capture systems are at the cutting edge of current photosynthetic research. Combined with molecular biology these new systems have the potential to extend and reshape traditional light harvesting capacity. Research related to harvesting far red light and inputting that energy into the photosynthetic process started with the discovery of chlorophyll *d* reaction center systems in photosynthetic bacteria (Allakhverdiev et al., 2016; Scheer, 2022), and has since been extended to chlorophyl *f* and infrared absorbing proteorhodopsin. In the other direction, in a spectral sense, the concept of “filling the green gap” makes intuitive sense as most major pigments absorb weakly in some portion of the 500-600 nm region, and this represents a significant portion of the total irradiance that reaches the Earth’s surface (Gundlach et al., 2009).

### 5.1 Infrared driven photosynthesis

Chlorophyll *d*, chlorophyll *f*, and infrared absorbing proteorhodopsin are examples in natural systems of photosynthetic activity under infrared (>700 nm) light. Chlorophyll d was first reported as a major pigment in the cyanobacterium *Acaryochloris marina* isolated from an ascidian (sea squirt) (Miyashita et al., 1996). It was later established that chlorophyll *d* was the central pigment in photosystem I, functioning at 750 nm (Hu et al., 1998). Recently, chlorophyll d was incorporated *in vitro* into the LHCII protein of *Spinacia oleracea* (spinach) by Elias et al. (Elias et al., 2021). The integration of chlorophyll *d* red shifted the LHCII absorption spectrum from 672 nm to 699 nm and the authors showed that the change did not lead to loss of function. Chlorophyll *f* was discovered more recently, and due to its red shift in absorption relative to other chlorophylls known to date, represents an opportunity to further expand the photosynthetic action spectrum (Chen et al., 2010). Currently, it has been expressed in *Synechococcus* sp. PCC 7002 light harvesting systems (Shen et al., 2019), and has replaced chlorophyll *a in vitro* in dinoflagellate LHC complexes (Hernández‐Prieto et al., 2022). Finally, in a novel approach, Chen et al. built-on work expressing proteorhodopsin in *Synechocystis* PCC 6803 by generating transformed strains with an infrared absorbing proteorhodopsin. This did not lead to improved growth, likely because of the relatively inefficient activity of the pump. However, it is an interesting addition to far red absorbing reaction centers, and acts as a proof of concept for integrating infrared absorbing proteorhodopsin into cyanobacterial photosystems (Chen et al., 2019).

### 5.2 Photosynthesis in the green gap

Extending the photosynthetic action spectrum into the 500-600 nm wavelength region has been proposed to lead to better solar energy capture. However, there is conflicting literature regarding whether green light can be effectively harvested. In 2009, Gundlach et al. reported that rhodamine red dyes can be attached to the LHCII of *Pisum sativum* (pea), and found the energy transfer of green light into the system was increased (Gundlach et al., 2009). However, as pointed out by another group more recently (Arsenault et al., 2020), the work by Gundlach was done *in vitro*, whereas green light can drive chlorophyll-chlorophyll energy transfers, which *in vivo* drives photosynthesis. This work was supported and inspired by a study published in 2009 on *Helianthus annuus* (sunflower) leaves, which showed that cells deeper in the leaf relied more heavily on green light to drive photosynthesis, rather than red or blue wavelengths (Terashima et al., 2009). However, as work in this emerging field has so far focused on plant LHCs and plant photosynthesis, it is not clear if it can be extended to microalgal or cyanobacterial cultures. It is possible that under certain growth conditions for microalgae and cyanobacteria, such as thin film bioreactors, the effect of green light may be overshadowed by red and blue light to the point of being insignificant.

More recently, two studies explored expanding the photosynthetic action spectrum using a mix of natural and synthetic pigments. Hancock et al. used self-assembling lipid vesicles with lipid linked chromophores and hydrophobic dyes attached or imbedded into the membrane, respectively. These pigments included plant LHCII, purple bacteria LH2 and the dye Cyanine 7 among others. The combination of these pigments led to near full absorption across the visible spectrum. By matching synthetic dyes with light harvesting systems based on complementary energy levels the authors were able to show light transfer outside the typical range of a given complex. Although this was purely an *in vitro* study, it effectively demonstrates that complex light harvesting systems can be augmented with a wider action spectrum (Hancock et al., 2022).

Complementing this idea is work done by Suarez et al. demonstrating that the Katushka fluorescent protein can be expressed in the chloroplast of *C. reinhardtii* leading to yellow light driven photosynthesis. This protein lowers the energy of yellow light photons (590 nm) to red light photons (649 nm) through fluorescence, converting the otherwise underused wavelengths into more photoactive radiation. They further showed that diminishing cell chlorophyll content enhanced the transgenic protein expression as the cells used the heterologous protein for light harvesting (Suarez et al., 2022). It should be noted that this study relied mostly on optical density measurements for monitoring growth and grew cells at very low light levels (30 µmol m^-2^s^-1^) in mixotrophic conditions. In addition, important parameters such as quantum yield were not provided. Regardless this represents a potential step forward in augmenting the photosynthetic system *in vivo*.

## 6 Conclusions

Despite major differences in physiology and large-scale growth, plants and algae share many parallels and research into the photosynthetic efficiency of one class of organisms often leverages ideas and results in another. In the context of improving photosynthetic efficiency, many fundamental studies are conducted on single celled cultures, whereas more applied studies are often conducted on plants. Due to natural photosynthetic evolution and physiology, these plant studies are often not backwards compatible when considering large scale microalgal or cyanobacterial cultivation. **Table 1** presents a summary of recent developments in photosynthetic research in several plant, microalgal, and cyanobacterial groups.

**Table 1:** Summary of genes and proteins reviewed in this paper.

Gene/Enzyme	Function	Citations	Cyano-bacteria	Micro-algae	Plants
Antiox	Antioxidant peptides capable of fusion directly to PSII D1 subunit	(Antonacci et al., 2021)	P	+	P
*atpA*	ATP synthase with reduced redox regulation	(Ungerer et al., 2018b)	+	P	P
*bkt*	Conversion of beta-carotene to astaxanthin or canthaxanthin	(Lin et al., 2019; Cazzaniga et al., 2022)	P	+	P
*cpc*	Phycocyanin synthesis	(Kirst et al., 2014; Sengupta et al., 2023)	+	–	–
COX	Cytochrome *c* oxidase that acts as an alternative electron acceptor to PSI	(Torrado et al., 2022)	+	–	–
CpSRP	Various chaperone enzymes for assembling light harvesting complexes (includes ALB and LTD)	(Baek et al., 2016; Jeong et al., 2018; Nymark et al., 2019; Rotasperti et al., 2022; Caddell et al., 2023; Krishnan et al., 2023)	–	+	+
*crtr-B*	Conversion of beta-carotene to astaxanthin or canthaxanthin	(Lin et al., 2019)	+	+	+
*cyn*28	Formation of protease FtsH complexes. Photodamage repair	(Fu et al., 2023)	P	+	P
Cyt P450	Cytochrome P450 that acts as an electron acceptor from PSI	(Santos-Merino et al., 2021; Torrado et al., 2022)	+	P	P
*dschyb*	Conversion of β-carotene to zeaxanthin	(Hu et al., 2021)	P	+	P
*flv*	Excess electron sink from PSI	(Tula et al., 2020; Smolinski et al., 2022)	+	P	+
*hpe1*	RNA signal recognition peptide related to nucleus-plastid communication	(Jin et al., 2016)	–	P	+
LHCSR	Excess excitation energy dissipation	(Barera et al., 2021)	–	+	P
LHC	Light capture and transfer to reaction centers (includes Lhcx)	(Buck et al., 2019; Krishnan et al., 2023)	–	+	+
*myb*	Transcription factor involved in LHC complex formation	(Agarwal et al., 2022)	–	+	P
*nblA*	Phycobilisome disassembly	(Carrieri et al., 2021)	+	–	–
*osbHLHqq11*	Transcription factor stimulating chlorophyll synthesis	(Jang et al., 2022)	–	–	+
*ppnK*	NAD^+^/ATP kinase with favorable kinetics	(Ungerer et al., 2018b)	+	P	P
PsbS, VDS, ZEP	PH dependent and VAZ cycle related NPQ	(Kromdijk et al., 2016)	–	+P	+
*rrl*	Phycobilisome rod-rod linker	(Sengupta et al., 2023)	+	–	–
*tpp*	Oxidative stress relief	(Rathod et al., 2023)	P	+	P
*ygl1*	Metabolite control in chlorophyll synthesis	(Mao et al., 2023)	P	P	+
*zmCRD*1	Chlorophyll Synthesis	(Xue et al., 2022)	P	P	+

+ indicates that a gene/enzyme was used in some organisms of that category, P represents that a gene/protein could be used in a category but was not represented in the literature presented in this paper, - indicates that use in that category is not feasible.

Included are recent publications exploring ideas and approaches to improving photosynthetic efficiency. Many of which are applicable across all three types of organisms, in particular the concept of heterologous pigment and transcription factor expression, and gene swapping. Several effective ideas relate to adding additional electron sinks or improving resistance to oxidative stress. Astaxanthin and canthaxanthin expression in particular has repeatedly been shown to improve photoprotection under high light, even in a foreign system. Another idea that proved successful in cyanobacteria is overexpressing a disassembly gene for phycobilisomes. This is a creative alternative approach to directed knockouts and could avoid the issue of unintended side effects due to multifunctional genes. It would be interesting to see similar work carried out in eukaryotic strains.

An area of this research that continues to produce contrasting results is the modification of pigmentation, reaction centers and NPQ mechanisms. Often modified strains with lower pigmentation show reduced growth, and several strains showing improved short term photosynthetic efficiency (F_v_/F_m_) do not achieve improved biomass productivities. An important idea that arises from this work is that changes in light harvesting pigments must be offset by an appropriate change in energy and electron flow, such as changes in NPQ or AEF. Similarly, energy transfer between both photosystems should be balanced by appropriate pigmentation and reaction center levels.

This idea will become more relevant as research continues into understanding far red and green light absorption and integration into ‘traditional’ photosystems. Much work remains to be done integrating novel pigments and dyes *in vivo*, however the absorption of additional yellow-green wavelengths and the utilization of the lower infrared wavelengths presents the balance of energy input and transport across the light systems as a double-sided issue. Overcoming this challenge is an important part of designing novel photosystems that allow biomass production from previously under used light.

One other point of comparison between plants and microalgae is in the context of industrial scale cultivation. Plant photosynthetic engineering focuses heavily on relevant staple plants that are currently used in industry. Much of microalgal engineering is focused on algal species that may not be viable for large scale cultivation, specifically because of a lack of robust growth under diel conditions which are often argued as advantages for large scale algal growth. Focusing on strains that grow robustly outdoors will become critical as demands on limited resources continue to increase. State-of-the-art agriculture will not be sufficient to supply the biomass and agricultural needs of the world’s population. To meet this challenge effectively researchers must be aware of the variety of photosystems already present, their advantages, and the tools that can be used to integrate and improve them.

## Author contributions

MP: Conceptualization, Writing – original draft, Writing – review & editing. GD: Conceptualization, Writing – original draft, Writing – review & editing.
